# Assisting hand gesture classification and rehabilitation assessment via sEMG and finger motion data

**DOI:** 10.3389/fbioe.2025.1751763

**Published:** 2026-01-08

**Authors:** Xiuxiu Yang, Lingfeng Zhang, Fukui Wu, Xinran Wei, Haifeng Huang, Jun Li, Tao Hu

**Affiliations:** 1 College of Intelligent Science and Engineering, Hubei Minzu University, Enshi, Hubei, China; 2 Sports Health and Collaborative Intelligent Engineering Research Center, Hubei Minzu University, Enshi, China; 3 Department of Electrical Engineering and Information Systems, Graduate School of Engineering, The University of Tokyo, Tokyo, Japan; 4 Hubei Engineering Research Center of Selenium Food Nutrition and Health Intelligent Technology, Enshi, Hubei, China

**Keywords:** classification, finger movement, machine learning algorithms, rehabilitation, sEMG

## Abstract

**Introduction:**

To address the lack of integrated and clinically applicable motion capture systems for hand function assessment, we developed a wearable device capable of simultaneously recording finger curvature and surface electromyography (sEMG) signals from both healthy individuals and patients with motor impairments.

**Methods:**

The dataset comprises 900 measurements of six predefined gestures collected from 15 participants using a six-channel sEMG motion-capture glove. Data were obtained through hospital-based field acquisition, ensuring clinical relevance and independence of the hardware–database framework. The recorded signals were processed using a Savitzky–Golay filter, followed by Short-Time Fourier Transform (STFT) for spectrogram generation. Multiple machine learning models, including SVM, LightGBM, and MLP, were employed for gesture classification.

**Results:**

Most models achieved over 90% precision on both cross-validation and test sets, demonstrating robust classification performance across different gesture types and subject conditions.

**Discussion:**

These results confirm that the proposed system maintains high recognition accuracy even in severely impaired subjects. The dataset presented here offers substantial value for gesture recognition research, rehabilitation assessment, and neuromuscular signal analysis.

## 1 Introduction

Finger motor dysfunction is commonly caused by neurological, muscular, or skeletal disorders, including stroke, spinal cord injury, neurodegenerative diseases (e.g., Parkinson’s disease), and trauma (Castelli et al., 2020). Such conditions disrupt neural signaling and muscular coordination, ultimately impairing fine motor control. While conventional clinical assessments remain standard practice, there is increasing interest in quantitative and objective evaluation technologies. These methods hold promise for early diagnosis, disease progression monitoring, and identification of condition-specific motor patterns (Nunes et al., 2022). Integrating such systems can automate traditional assessment procedures, provide objective biomarkers, and establish a closed-loop evaluation framework that improves diagnostic consistency and reduces clinician-dependent variability.

Recent developments in quantitative assessment methods for finger motor dysfunction can be broadly divided into two biosignal-driven approaches: surface electromyography (sEMG) for neuromuscular intent decoding and finger joint kinematics for motion tracking. sEMG enables non-invasive identification of motor intention by capturing muscle electrophysiological activity (Sadikoglu et al., 2017), whereas kinematic sensors supplement this by quantifying overt joint movement. These advancements align with the rapid progress in neurorobotics, encompassing areas such as motion intent decoding and haptic feedback systems (Li et al., 2023), as well as the assessment of patient muscle activity to enhance human-robot interaction in rehabilitation (Zhang et al., 2025; Cao et al., 2025). Prior studies have demonstrated that multi-objective optimization improves the robustness and accuracy of sEMG-based myoelectric control (Shaikh et al., 2024). Recent innovations include high-density electrode arrays for enhanced spatial resolution (Yeung et al., 2024), durable flexible sEMG sensors (Gao et al., 2024), and adaptive processing algorithms that mitigate motion artifacts and electrode displacement (Li et al., 2021). Meanwhile, multiphysics sensing technologies—such as FBG-based strain sensors for scalable finger-angle tracking (Kim et al., 2020) and dual IMU-EM frameworks for micro-gesture recognition (Liang et al., 2021)—further advance the precision of joint motion capture.

Existing methods for evaluating hand function commonly rely on either sEMG or kinematic signals (Suo et al., 2024), (De Smedt et al., 2016), yet each modality presents inherent limitations. sEMG is highly susceptible to noise interference and cannot adequately represent movement dynamics, whereas kinematic data alone fail to capture underlying neural control. Although recent studies indicate that multimodal fusion offers a more comprehensive assessment, its clinical translation remains challenging (Duan et al., 2024; Samui et al., 2023). To address these issues, we introduce a synchronised sEMG–kinematic acquisition system together with a multimodal learning framework capable of achieving robust gesture recognition across varying levels of motor impairment.

**FIGURE 1:**
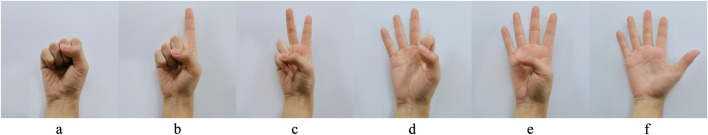
Measure the standard of action **(a)**: right hand fist, indicating “0” **(b)**; extend the index finger and bend the other four fingers to indicate “1” **(c)**; raise your index and middle fingers to indicate “2” **(d)**; the thumb and index finger are matched, as if indicating “OK” gesture, and the other three fingers are raised, indicating “3” **(e)**; the thumb is bent to the palm center, and the other four fingers are straight, indicating “4” **(f)**; the fingers are extended and the palm is facing outward, indicating “5”.

These innovations tackle the scarcity of synchronized multimodal data and reduce reliance on healthy subject models. We developed a thin-film resistive sensor integrated into a fiber-woven glove with six high-density sEMG channels, enabling simultaneous muscle and joint motion capture without spatiotemporal mismatch. To support real-world diagnosis, data were collected from 10 patients with myelitis, cerebral infarction, and cerebral hemorrhage during standardized gestures (Figure 1). Unlike public datasets focused on healthy subjects (Mukhopadhyay and Samui, 2020; Ozdemir et al., 2022; Furmanek et al., 2022; Salter et al., 2024). Our dataset incorporates impaired patients, improving clinical relevance. Meanwhile, multimodal sEMG fusion continues to demonstrate superiority in robustness and decoding performance, as shown in EMG–vision hybrid inference for prosthetic grasp intention (Zandigohar et al., 2024). Consistent with this trend, our multimodal fusion of sEMG and finger movement achieved 93.6% average precision in classifying motor neuron lesions and severity, outperforming sEMG-only (87.8%) or motion-only (87.8%) methods. The innovation highlights of our work are summarized as follows:

1. Novel Wearable System Integration: Developed a low-cost multichannel sEMG acquisition module integrated into a flexible data glove, enabling synchronous and multimodal capture of muscle activity and kinematic finger movement signals.
2. Clinically-Grounded Multimodal Dataset: Constructed a unique multimodal dataset comprising sEMG and motion data from 15 participants with varying degrees of hand motor function—including healthy individuals and patients with severe, moderate, and mild impairments—ensuring broad clinical relevance and ecological validity.
3. Robust and Reproducible Evaluation Framework: Consistent >90% accuracy across conventional ML models under cross-validation and independent testing confirms the dataset’s reliability for clinical rehabilitation, even in severe cases.

## 2 Methods

A six-channel sEMG sensor and a motion capture glove were used to collect gesture data from 15 participants, including healthy individuals and people with movement disorders. The sampling rate of sEMG signals is 225 Hz, and the data are preprocessed by the Savitzky–Golay filter and STFT for subsequent algorithm analysis.

### 2.1 Data acquisition

All experimental protocols involving human participants in this study were reviewed and approved by the Medical Ethics Committee of Hubei University for Nationalities [Ethics Approval Number: (2025) 05]. Before the study commenced, the researchers explained the study objectives in detail to all participants or their legal guardians to ensure full understanding of the research. All participants voluntarily signed written informed consent forms. During the study, participants’ personal identifying information was replaced with Subject IDs instead of real names.

The dataset consists of 15 participants, including healthy individuals and patients with varying degrees of hand motor impairment, rated from 0 (no movement) to 4 (normal function) (Table 1). Motor ability was assessed based on observable voluntary control: 0 indicates a complete loss of voluntary movement with no observable joint motion; 1 indicates slight voluntary movement that can perform the intended action, but with poor coordination and noticeably slow execution; 2 represents the ability to perform simple movements with limited strength, amplitude, and precision; 3 denotes the ability to carry out most daily movements with near-normal function, though mild deficits may still appear in fine or rapid actions; and 4 corresponds to normal, well-coordinated movement without observable impairment.

**TABLE 1:** Motor function types and scores for subjects.

Subject ID	Gender	Motor function type	Motor function score
1	Female	Cerebral hemorrhage	0
2	Female	Myelitis	0
3	Male	Hemiplegia	0
4	Male	Cerebral infarction	0
5	Male	Cerebral infarction	1
6	Male	Cerebral infarction	1
7	Male	Cerebral infarction	2
8	Female	Cerebral infarction	2
9	Male	Cerebral infarction	3
10	Female	Healthy	4
11	Female	Healthy	4
12	Male	Healthy	4
13	Male	Healthy	4
14	Male	Healthy	4

**FIGURE 2:**
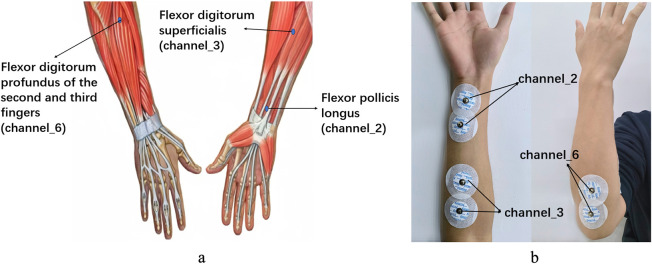
Muscle and sEMG patch placement; the location of **(a)** the relevant muscles of the right upper limb and their corresponding channels (Samui et al., 2023), and **(b)** the location of the sEMG patch on the arm.

Data were collected at the Affiliated Hospital of Hubei University for Nationalities and Laifeng County People’s Hospital using a six-channel sEMG sensor and a motion-capture glove. Participants performed six standardised gestures (0–5, Figure 1) while sEMG signals and finger-curvature data were recorded. Electrodes were placed over three target muscles—flexor pollicis longus (thumb), flexor digitorum superficialis (fingers 2–5) and flexor digitorum profundus (fingers 2–3)—following established placement guidelines (Ferrante et al., 2024). Pearson correlation analysis (Table 2) showed that channels 2, 3 and 6 had the strongest association with finger flexion, and were therefore used for further analysis. Electrode positions are shown in Figure 2.

**TABLE 2:** Pearson correlation between fingers and channels.

Channel	Pinky	Ring finger	Middle finger	Fore finger	Thumb
Channel 1	0.023	−0.043	−0.023	−0.055	0.059
Channel 2	0.381	0.336	0.319	0.308	0.507
Channel 3	0.487	0.386	0.397	0.381	0.510
Channel 4	0.040	−0.097	−0.090	−0.110	0.179
Channel 6	0.265	0.229	0.200	0.187	0.309

All participants performed six predefined gestures after viewing the demonstration video. During execution, they maintained a relaxed posture with forearms resting naturally on the table or bed to minimise postural interference. Healthy subjects completed the movements independently based on visual cues, while testers provided verbal instructions and demonstrations for subjects with motor impairments to ensure correct understanding and maximise task completion.

### 2.2 Acquisition equipment

**FIGURE 3:**
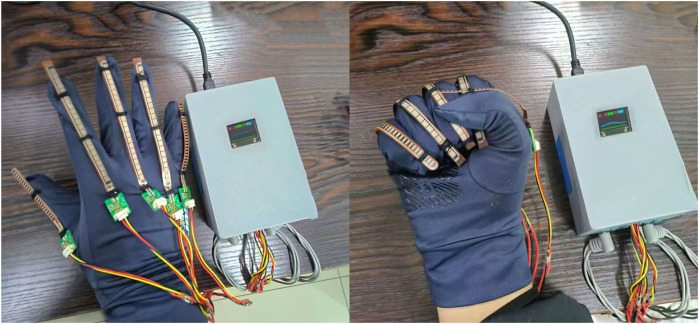
The system comprises a sensor-equipped glove linked to a compact processing unit. The glove captures hand postures—from extended to clenched positions—while the unit integrates a multi-channel EMG acquisition module and a microcontroller (the left image). Acquired signals are processed in real-time, with muscle activity displayed on the built-in screen (the right image).

**FIGURE 4:**
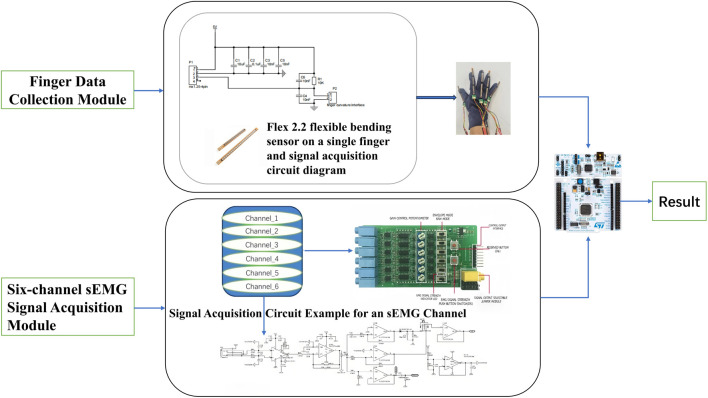
The finger movement acquisition system consists of a glove-integrated bending sensor module and a six-channel sEMG module. The glove module uses Flex 2.2 sensors to capture finger flexion, while the sEMG module acquires muscular activity signals. Both signals are processed by a central unit to produce the final output.

Figure 3 shows the equipment used for this data collection. Figure 4 shows the data transmission process of the data acquisition device, which is based on the STM32F103VET6 microcontroller (Tong et al., 2024) and integrates the finger curvature sensor and the six-channel sEMG acquisition module (Sapsanis et al., 2013). The finger curvature sensor acquires finger movement data by detecting resistance changes (Yin et al., 2018), while the sEMG acquisition module records EMG signals from forearm muscles through six electrodes with a sampling rate of 225 Hz. All data are transmitted through the serial port and saved as CSV files for subsequent processing and analysis.

### 2.3 Signals pre-processing

A preliminary cleaning step was applied to remove erroneous entries, missing values, and outliers, thereby reducing noise and improving data reliability. The cleaned data were then standardised and stored in CSV format to facilitate subsequent processing and analysis.

Following data cleaning, all signals were smoothed using the Savitzky–Golay filter. Each dataset file contains eight time-series channels, each corresponding to one signal stream. All channels underwent the same filtering procedure to retain key waveform characteristics while suppressing high-frequency noise. The mathematical expression of the Savitzky–Golay filter is shown in Equation 1 (Lei, 2014).

$$y\left[n\right]=\sum_{k=-M}^{M}c_{k}\cdot x\}\left[n+k\right]$$ (1)

In Equation 1, *2M+1* is the filter window length (optimised to 101 in this study, *M = 50*). $c_{k}$ denotes the coefficients obtained from least-squares fitting of a p-order polynomial (optimised to 4th order). Zero-padding was applied at both signal edges to prevent boundary artefacts and ensure valid indexing. This filter is a technique used for data smoothing that creates a smooth approximate version of the original data by performing a local polynomial regression on it (Cai and Tang, 2011). The Savitzky–Golay filter smooths signals by fitting local polynomial regressions, thereby preserving waveform characteristics while reducing noise (Cai and Tang, 2011).

**FIGURE 5:**
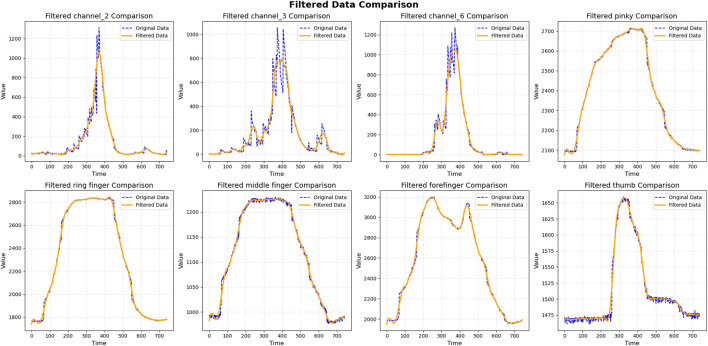
Comparison of selected channels before and after filtering for subject 0 is shown herein. Eight subplots display original versus filtered signals across three channels and five finger movements. The filtered data (orange lines) exhibit substantially reduced noise compared to the original signals (blue dashed lines), while preserving underlying signal morphology.

**FIGURE 6:**
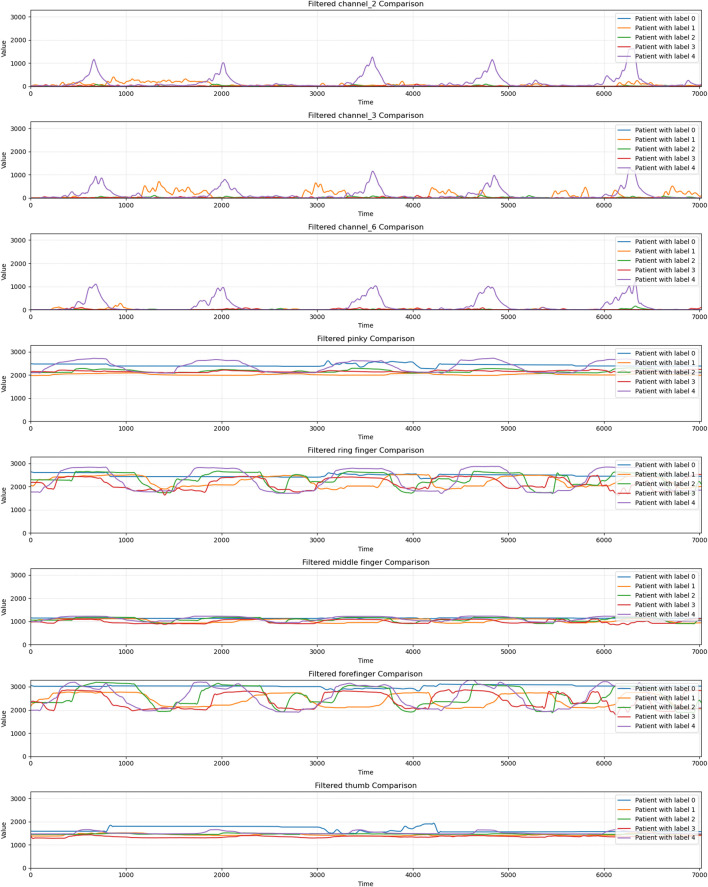
Comparison of filtered finger curvature and muscle electrical data from action 0 between healthy individuals and patients with hand injuries.

Figure 5 presents a comparison of raw and filtered signals for gesture 0 from Subject 9. As shown in Figure 5, the filtered data exhibit visibly smoother trajectories with reduced high-frequency fluctuations, particularly near peak regions. These results, further supported by Figure 6, confirm that the Savitzky–Golay filter effectively suppresses noise while maintaining the essential temporal structure of both finger-curvature and sEMG channels.

As shown in Figure 6, subjects with motor function level 4 exhibit the strongest hand mobility, characterised by clear waveform fluctuations in both finger-curvature and sEMG channels, indicating active neuromuscular engagement. Individuals at level 3 retain relatively good coordination and movement amplitude, with pronounced activity in the thumb and index finger. In contrast, subjects with levels 1–2 show markedly reduced movement, reflected by weaker and less stable signal variations. The level 0 subject displays almost no detectable hand motion, with minimal fluctuations across all channels, indicating near-complete functional loss.

From these analysis results, it can be seen that the degree of impairment of hand function is closely related to the fluctuation of channel and finger data. The more frequent and larger the fluctuation, the better the motor function of the hand. In contrast, hand function may be limited or lost. These data can be used to help assess and track recovery from hand function in patients and provide a basis for rehabilitation treatment.

### 2.4 Spectrogram rendering based on STFT

The Short-Time Fourier Transform (STFT) was applied to perform time-frequency analysis on the processed data. The STFT formula (Wang et al., 2020) is given by Equation 2.

$$X\left[m,k\right]=\sum_{\left(n=0\right)}^{\left(N_{win}-1\right)}x\left[m\cdot N_{hop}+n\right]\cdot w\left[n\right]\cdot e^{\left(-j2\pi kn/N_{win}\right)}$$ (2)

In Equation 2, $N_{win}$ = 64 is the number of window samples, $N_{hop}$ = 32 is the window step size (50% overlap), $w\left[n\right]$ is a Hamming window (to reduce spectral leakage). $X\left[m,k\right]$ is a complex coefficient, and the square of its magnitude is used for the spectrogram modelling.

**FIGURE 7:**
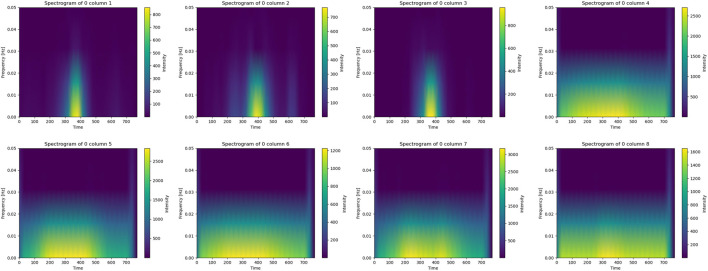
Time-frequency spectrograms of eight distinct data columns from Subject 9 (derived from acquired and filtered signals) are shown. The horizontal axis represents time, the vertical axis frequency, and spectral intensity is indicated by a color gradient (purple for lowest, yellow for highest).

Time–frequency features are widely used to reduce limb-position interference in sEMG recognition tasks (Khushaba et al., 2014). Since the raw samples vary in length, direct processing is cumbersome; therefore, STFT was applied to convert the signals into spectrograms with unified dimensions. Compared with the original waveform, spectrograms provide a clearer depiction of temporal frequency variations, reduce noise interference, and enhance model recognition performance (Xie et al., 2012). As shown in Figure 7, spectrograms were generated using matplotlib, with time and frequency as axes and colour intensity representing spectral energy, enabling intuitive observation of dynamic signal changes.

### 2.5 Effect of preprocessing parameters

We evaluated the impact of the preprocessing parameters on classification performance. The effects of the STFT and Savitzky–Golay filter configurations are detailed in Tables 3, 4 respectively.

**TABLE 3:** Effect of STFT parameters on classification accuracy.

Nperseg	Noverlap	Cv acc	Test acc
64	16	0.8842	0.9167
64	32	0.9274	0.9167
64	48	0.8731	0.9444
128	32	0.8088	0.8556
128	64	0.8480	0.8777
128	96	0.8591	0.9000
256	64	0.7559	0.7611
256	128	0.7796	0.7944
256	192	0.8242	0.8500

**TABLE 4:** Effect of savitzky-golay filter parameters on classification accuracy.

Window length	Polyorder	Cv acc	Test acc
11	2	0.8870	0.9111
11	3	0.8731	0.9111
11	4	0.8731	0.9111
51	2	0.8661	0.9111
51	3	0.8828	0.9388
51	4	0.8926	0.9056
101	2	0.9010	0.9000
101	3	0.8982	0.9111
101	4	0.9177	0.9222

#### 2.5.1 STFT parameter variation

As shown in Table 3, the STFT parameters *nperseg* and *noverlap* have a clear impact on model performance. The optimal cross-validation accuracy (0.9274) was obtained with *nperseg = 64* and *noverlap = 32*, while the highest test accuracy (0.9444) was achieved at *nperseg = 64* and *noverlap* = *48*. Overall, larger segment lengths tended to reduce accuracy, likely due to the loss of temporal resolution.

#### 2.5.2 Savitzky–Golay filter parameter variation

As shown in Table 4, the Savitzky–Golay filter parameters (window length and polyorder) also had a notable effect on model performance. The best results were obtained with a window length of 101 and a polynomial order of 4, yielding cross-validation and test accuracies of 0.9177 and 0.9222, respectively. This is consistent with expectations—larger windows enhance smoothing, while a moderate polynomial order preserves key signal features.

In summary, we found the optimal performance with an STFT configuration of *nperseg = 64*, *noverlap = 32* and an SG filter configuration of *window length = 101, polyorder = 4*.

## 3 Experiment

Experiments were conducted on a Windows 10 (version 22H2) platform with an Intel Core i3-9100 CPU (3.60 GHz). All algorithms were implemented in Python 3.8. Five machine learning models—KNN, SVM, MLP, LightGBM, and Random Forest—were used to evaluate the performance of our self-built dataset.

### 3.1 Technical validation

**FIGURE 8:**
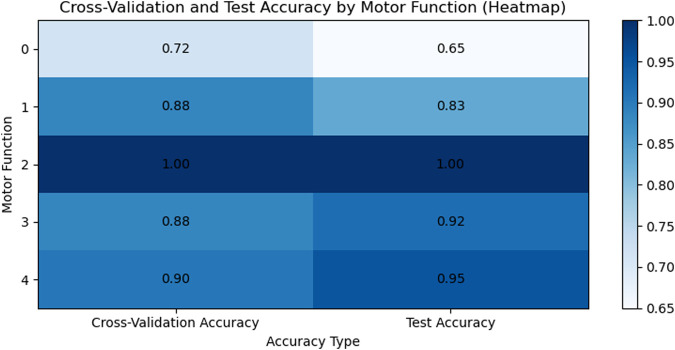
The heat map illustrates the cross-validation and test set accuracy of motor function classification using the KNN model.

**FIGURE 9:**
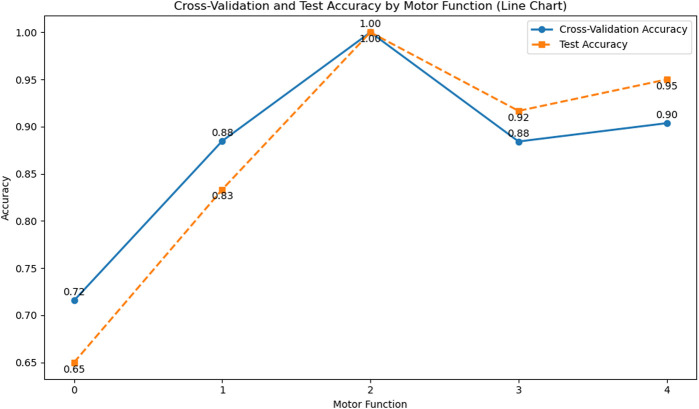
Line graph of cross-validation accuracy and test set accuracy of motor function on the KNN model. In the legend, the blue solid line represents cross-validation accuracy, and the orange dotted line represents test set accuracy.

We used the KNN model to classify subjects with different levels of motor impairment, with the classification performance for each level shown in Figures 8, 9. Figure 8 displays the accuracy heatmaps corresponding to five impairment levels, while Figure 9 illustrates the results in line-chart form. As shown, the KNN model achieved the highest accuracy for subjects with motor function scores of 3 and 4.

However, clear differences in signal properties and model performance were observed across impairment levels. Subjects with a motor function score of 0 showed almost no voluntary movement and produced weak, unstable sEMG activity, resulting in highly similar patterns across gestures and poor classification accuracy. In contrast, subjects with higher function levels generated more distinguishable signals, leading to stronger model performance. Overall, these results indicate that sEMG remains effective for differentiating motor function states, even among individuals with notable impairment.

### 3.2 Model construction

We used five machine learning models (KNN (Zhang and Li, 2021), SVM (Pisner et al., 2020), MLP (Taud et al., 2018), LightGBM (Sun et al., 2018), and Random Forest (Liu et al., 2012)) to test the classification of our self-built dataset.

**FIGURE 10:**
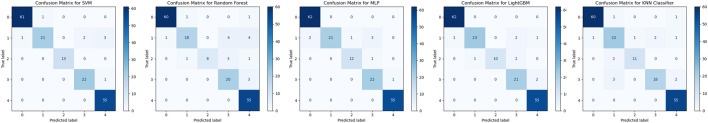
Confusion matrices for the five models (SVM, Random Forest, MLP, LightGBM, and KNN) are presented, each illustrating the classification performance of true labels versus predicted labels. Each row represents a true label, and each column represents a predicted label.

Figure 10 shows the confusion matrices of the five classification models. While the models differ in classification performance, most exhibit relatively low misclassification rates, reflecting good overall recognition performance.

Table 5 presents the performance evaluation of five models—SVM, Random Forest, MLP, LightGBM, and KNN—on both multi-modal fusion sensor and single-sensor test sets. The F1-score, defined as the harmonic mean of precision and recall, reflects the overall effectiveness of a model in classification tasks (Lam et al., 2023). MCC takes into account true and false positives and negatives, making it particularly suitable for evaluating imbalanced datasets (Itaya et al., 2024). Cohen’s Kappa measures classification consistency by accounting for random agreement (McHugh, 2012). Recall evaluates the ability of the model to identify positive instances, that is, the proportion of actual positives correctly captured (Saito and Rehmsmeier, 2015).

**TABLE 5:** Performance evaluation results of five models on multi-modal fusion sensors and single sensor test sets.

Model	Sensor type	Test recall	Test F1-score	Matthews correlation coefficient	Cohen’s kappa score
SVM	sEMG + Flex sEMG Flex	0.956 0.917 0.827	0.954 0.916 0.833	0.941 0.888 0.824	0.940 0.888 0.775
Random forest	sEMG + Flex sEMG Flex	0.894 0.811 0.844	0.891 0.790 0.821	0.860 0.746 0.796	0.857 0.736 0.784
MLP	sEMG + Flex sEMG Flex	0.956 0.872 0.900	0.954 0.871 0.895	0.941 0.830 0.868	0.940 0.828 0.864
Light GBM	sEMG + Flex sEMG Flex	0.950 0.911 0.922	0.949 0.912 0.919	0.933 0.881 0.896	0.932 0.881 0.894
KNN	sEMG + Flex sEMG Flex	0.922 0.879 0.833	0.921 0.878 0.824	0.896 0.874 0.775	0.895 0.836 0.771

As illustrated in Table 5, the multi-sensor fusion strategy achieves the highest performance in all models. Among these, SVM and MLP deliver the best classification results, demonstrating strong robustness, especially in handling imbalanced data. LightGBM follows closely with competitive performance, while KNN and Random Forest show moderate results. These findings underscore the importance of model selection in improving classification accuracy, particularly in tasks that require high recall and consistency, for which SVM and MLP emerge as preferable options.

**FIGURE 11:**
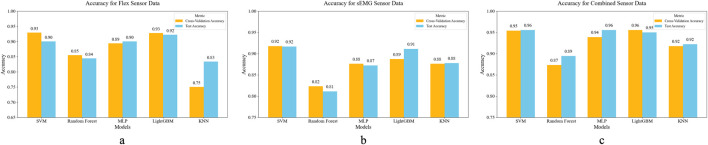
**(a)** Accuracy for Flex Sensor Data **(b)** Accuracy for sEMG Sensor Data **(c)** Accuracy for Combined Sensor Data. Comparison of classification accuracy of each model under different sensor configurations.

Finger movement data and electromyography signals from different subjects performing specified actions were input into five machine learning models. Figure 11 illustrates the cross-validation accuracy and test accuracy of the five machine learning models under different sensor configurations. The cross-validation accuracy reflects the stability of the model in the training set, while the test accuracy evaluates its performance on unseen data. The results indicate that the multi-sensor fusion approach yields the best performance across all models. Specifically, SVM, LightGBM, and MLP achieve high performance in both cross-validation and test sets, each exceeding 95% accuracy. The KNN model shows a slight decrease in the test set, but still maintains high accuracy. In contrast, Random Forest performs relatively poorly in this task, which may be attributed to its sensitivity to high-dimensional feature spaces.

To evaluate performance on an external dataset, we conducted comparative experiments, as shown in Table 6. We used the Mendeley sEMG Gesture dataset (Ozdemir et al., 2022), selecting electromyography data corresponding to six standard gesture categories from 15 subjects as experimental samples. In model training and testing, for five machine learning classifiers, the same preprocessing pipeline as our self-collected dataset, with parameters adjusted according to the dataset characteristics. As seen from the tabular data, these classifiers performed well on the Mendeley dataset, achieving an average accuracy of 87.34%. This suggests that the adopted preprocessing and classification strategy remains effective when applied to another publicly available sEMG dataset.

**TABLE 6:** Comparison of classificationaccuracy on oudataset and the others datasets.

Dataset	SVM	Random forest	MLP	Light GBM	KNN
Mendeley	0.967	0.867	0.844	0.911	0.778
Our dataset	0.956	0.894	0.956	0.950	0.922

The data acquisition glove used in this study is made of elastic knitted fabric, offering a comfortable fit for most adult users, though performance may decline in individuals with severe muscle atrophy. Durability tests indicate that the integrated flexible sensors withstand up to one million bending cycles before noticeable performance degradation occurs, suggesting suitability for regular household or community use. However, the sEMG electrode pads may loosen due to perspiration and require timely replacement to maintain data continuity. Tests performed outside controlled hospital environments also revealed that noise and interference increase under less stable conditions. Therefore, measurements are recommended in quiet settings with steady posture for optimal signal quality.

### 3.3 Limitations

This study presents a self-built multimodal dataset consisting of sEMG and finger-movement signals, demonstrating its value in hand function assessment. Nonetheless, several limitations exist. First, the sample size is relatively small (15 subjects) with an uneven distribution of impairment types, and variations in neuromuscular patterns across different pathologies may affect model reliability. Therefore, larger and more diverse clinical cohorts are required to further validate the findings. Second, although the textile glove and adhesive electrodes provide good wearability, fit instability may occur in patients with muscle atrophy, and long-term use can reduce sensor sensitivity. Signal quality also fluctuates with sweat, posture, and other external factors. Future work will address these issues by expanding participant diversity, improving glove durability, and enhancing robustness against interference.

### 3.4 Conclusion and future work

This work presents a device capable of synchronously acquiring sEMG and finger-movement signals, together with a dataset collected from subjects with different levels of hand motor function. The multimodal data provide a more complete representation of neuromuscular activity and physical movement, supporting applications in gesture recognition, rehabilitation assessment, and user identification. Benchmark experiments demonstrate that the dataset is reliable and suitable for multiple analytical tasks.

Future efforts will focus on increasing the participant pool, enriching impairment categories, and integrating additional sensing modalities. Improvements in glove durability and anti-interference performance will also be explored. These developments are expected to further advance research in motor-function assessment and contribute to the design of intelligent assistive rehabilitation systems.

## Data Availability

The original contributions presented in the study are included in the article/Supplementary Material, further inquiries can be directed to the corresponding author.

## References

[B1] Cai T. J. Tang H. (2011). A review of the least squares fitting principle of the savitzky-golay smoothing filter. Digit. Commun. 38 (01), 63–68.

[B2] Cao Y. Ma S. Zhang M. Liu J. Huang J. Zhang Z. Q. (2025). Neuro-fuzzy musculoskeletal model-driven assist-as-needed control *via* impedance regulation for rehabilitation robots. IEEE Trans. Fuzzy Syst. 33, 4277–4288. 10.1109/tfuzz.2025.3611266

[B3] Castelli G. Desai K. M. Cantone R. E. (2020). Peripheral neuropathy: evaluation and differential diagnosis. Am. Fam. Physician 102 (12), 732–739. 33320513

[B4] De Smedt Q. Wannous H. Vandeborre J.-P. (2016). “Skeleton-based dynamic hand gesture recognition,” in Proceedings of the IEEE conference on computer vision and pattern recognition (CVPR) workshops.

[B5] Duan J. W. Xiong J. Q. Li Y. H. Ding W. P. (2024). Deep learning based multimodal biomedical data fusion: an overview and comparative review. Inf. Fusion 112, 102536. 10.1016/j.inffus.2024.102536

[B6] Ferrante L. Sridharan M. Zito C. Farina D. (2024). Toward impedance control in human–machine interfaces for upper-limb prostheses. IEEE Trans. Biomed. Eng. 71 (9), 2630–2641. 10.1109/TBME.2024.3384340 38564343

[B7] Furmanek M. P. Mangalam M. Yarossi M. Lockwood K. Tunik E. (2022). A kinematic and EMG dataset of online adjustment of reach-to-grasp movements to visual perturbations. Sci. Data 9 (23), 23. 10.1038/s41597-021-01107-2 35064126 PMC8782875

[B8] Gao Z. Q. Wang F. Bai Y. J. (2024). Flexible surface electromyography electrode based on fiber skeleton reinforcement. China Plast. 38 (7), 68. 10.19491/j.issn.1001-9278.2024.07.012

[B9] Itaya Y. Tamura J. Hayashi K. Yamamoto K. (2024). Asymptotic properties of matthews correlation coefficient. arXiv Prepr. 10.48550/arXiv.2405.12622 PMC11702320 39682035

[B10] Khushaba R. N. Takruri M. Miro J. V. Kodagoda S. (2014). Towards limb position invariant myoelectric pattern recognition using time-dependent spectral features. Neural Netw. 55, 42–58. 10.1016/j.neunet.2014.03.007 24721224

[B11] Kim J. S. Kim B. K. Jang M. Kang K. Kim D. E. Ju B.-K. (2020). Wearable hand module and real-time tracking algorithms for measuring finger joint angles of different hand sizes with high accuracy using FBG strain sensor. Sensors 20 (7), 1921. 10.3390/s20071921 32235532 PMC7181016

[B12] Lam K. F. Y. Gopal V. Qian J. (2023). Confidence intervals for the F1 score: a comparison of four methods. arXiv Preprint. 10.48550/arXiv.2309.14621

[B13] Lei L. P. (2014). Curve smoothing and denoising based on the Savitzky-golay algorithm. Comput. Inf. Technol. 22 (05), 30–31. 10.19414/j.cnki.1005-1228.2014.05.011

[B14] Li Q. Zhang A. Y. Li Z. L. Wu Y. (2021). Improvement of EMG pattern recognition model performance in repeated uses by combining feature selection and incremental transfer learning, Front. Neurorobot. 15. 10.3389/fnbot.2021.699174 34194311 PMC8236575

[B15] Li M. Secco E. L. Zheng Y. Dai C. Xiong P. Xu G. (2023). Advances in haptic feedback for neurorobotics applications. Front. Neurosci. 17, 1189749. 10.3389/fnins.2023.1189749 37113146 PMC10126486

[B16] Liang C. Yu C. Qin Y. Wang Y. T. Shi Y. C. (2021). DualRing: enabling subtle and expressive hand interaction with dual IMU rings. Proc. ACM Interact. Mob. Wearable Ubiquitous Technol. 5 (3), 1–27. 10.1145/3478114

[B17] Liu Y. Wang Y. Zhang J. (2012). “New machine learning algorithm: random forest,”. Information computing and applications. Editors Liu B. Ma M. Chang J. (Springer), 7473, 281–292. 10.1007/978-3-642-34062-8_32

[B18] McHugh M. L. (2012). Interrater reliability: the kappa statistic. Biochem. Medica 22 (3), 276–282. 10.11613/bm.2012.031 23092060 PMC3900052

[B19] Mukhopadhyay A. K. Samui S. (2020). An experimental study on upper limb position invariant EMG signal classification based on deep neural network. Biomed. Signal Process. Control 58, 101669. 10.1016/j.bspc.2019.101669

[B20] Nunes A. S. Kozhemiako N. Stephen C. D. Schmahmann J. D. Khan S. Gupta A. S. (2022). Automatic classification and severity estimation of ataxia from finger tapping videos. Front. Neurology 12, 795258. 10.3389/fneur.2021.795258 35295715 PMC8919801

[B21] Ozdemir M. A. Kisa D. H. Guren O. Akan A. (2022). Dataset for multi-channel surface electromyography (sEMG) signals of hand gestures. Data in brief. Amsterdam, The Netherlands: Elsevier Inc., 41, 107921. 35198693 10.1016/j.dib.2022.107921 PMC8844426

[B22] Pisner D. A. Schnyer D. M. (2020). “Support vector machine,” in Machine learning. Editors Mechelli A. Vieira S. (Academic Press), 101–121. 10.1016/B978-0-12-815739-8.00006-7

[B23] Sadikoglu F. Kavalcioglu C. Dagman B. (2017). Electromyogram (EMG) signal detection, classification of EMG signals and diagnosis of neuropathy muscle disease. Procedia Comput. Sci. 120, 422–429. 10.1016/j.procs.2017.11.259

[B24] Saito T. Rehmsmeier M. (2015). The precision-recall plot is more informative than the ROC plot when evaluating binary classifiers on imbalanced datasets. PloS One 10 (3), e0118432. 10.1371/journal.pone.0118432 25738806 PMC4349800

[B25] Salter S. Warren R. Schlager C. Spurr A. Han S. Bhasin R. (2024). emg2pose: a large and diverse benchmark for surface electromyographic hand pose estimation. Adv. Neural Inf. Process. Syst. 37, 55703–55728. 10.52202/079017-1770

[B26] Samui S. Garai S. Ghosh A. Mukhopadhyay A. K. (2023). “Heterogeneous stacked ensemble framework for surface electromyography signal classification,” in Proceedings of the international conference on pattern recognition and machine intelligence (PReMI) (Kolkata, India: Springer), 681–689. 10.1007/978-3-031-45170-6_70

[B27] Sapsanis C. Georgoulas G. Tzes A. (2013). “EMG based classification of basic hand movements based on time-frequency features,” in 21st mediterranean conference on control and automation, 716–722. 10.1109/MED.2013.6608802 24111045

[B28] Shaikh A. A. Mukhopadhyay A. K. Poddar S. Samui S. (2024). Towards robust and accurate myoelectric controller design based on multi-objective optimization using evolutionary computation. IEEE Sensors J. 24 (2), 1234–1245. 10.1109/JSEN.2023.3347949

[B29] Sun X. L. Liu M. X. Sima Z. Q. (2018). A novel cryptocurrency price trend forecasting model based on LightGBM. Finance Res. Lett. 32, 191–198. 10.1016/j.frl.2018.12.032

[B30] Suo M. R. Zhou L. N. Wang J. Z. Huang H. G. Zhang J. Sun T. Z. (2024). The application of surface electromyography technology in evaluating paraspinal muscle function. Diagnostics 14 (11), 1086. 10.3390/diagnostics14111086 38893614 PMC11172025

[B31] Taud H. Mas J. (2018). “Multilayer perceptron (MLP),” in Geomatic approaches for modeling land change scenarios. Editors Camacho Olmedo M. Paegelow M. Mas J. F. Escobar F. (Springer), 451–455. 10.1007/978-3-319-60801-3_27

[B32] Tong Z. Zhang J. Zhang W. (2024). Hardware and software design of programmable medium and high-speed data acquisition (DAQ) board of fiber optic signal for partial discharge acquisition. Electronics 13 (11), 2176. 10.3390/electronics13112176

[B33] Wang X. B. Ying T. Tian W. (2020). “Spectrum representation based on STFT,” in 2020 13th international congress on image and signal processing, BioMedical engineering and informatics (CISP-BMEI), 435–438. 10.1109/CISP-BMEI51763.2020.9263516

[B34] Xie H. Lin J. Lei Y. G. Liao Y. H. (2012). Fast-varying AM–FM components extraction based on an adaptive STFT. Digit. Signal Process. 22 (5), 896–903. 10.1016/j.dsp.2012.02.007

[B35] Yeung D. Negro F. Vujaklija I. (2024). Adaptive HD-sEMG decomposition: towards robust real-time decoding of neural drive. J. Neural Eng. 21 (2), 026012. 10.1088/1741-2552/ad33b0 38479007

[B36] Yin S. R. Yang J. Qu Y. K. Liu W. J. Guo Y. F. Liu H. T. (2018). “Research on gesture recognition technology of data glove based on joint algorithm,” in 2018 international conference on mechanical, electronic, control and automation engineering, 41–50. 10.2991/mecae-18.2018.8

[B37] Zandigohar M. Han M. Sharif M. Günay S. Y. Furmanek M. P. Yarossi M. (2024). Multimodal fusion of EMG and vision for human grasp intent inference in prosthetic hand control. Front. Robotics AI 11, 1312554. 10.3389/frobt.2024.1312554 38476118 PMC10927746

[B38] Zhang S. C. Li J. Y. (2021). KNN classification with one-step computation. IEEE Trans. Knowl. Data Eng. 35 (3), 2711–2723. 10.1109/tkde.2021.3119140

[B39] Zhang X. Cao Y. Huang J. Liu J. Zhang Z. Q. (2025). A systematic review of spiking neural networks for human-robot interaction in rehabilitative wearable robotics. IEEE Trans. Cognitive Dev. Syst. 1–15. 10.1109/tcds.2025.3599432

